# Challenges in diagnosing scrub typhus among hospitalized patients with undifferentiated fever at a national tertiary hospital in northern Vietnam

**DOI:** 10.1371/journal.pntd.0007928

**Published:** 2019-12-05

**Authors:** Shungo Katoh, Ngo Chi Cuong, Sugihiro Hamaguchi, Pham Thanh Thuy, Do Duy Cuong, Le Kim Anh, Nguyen Thi Hien Anh, Dang Duc Anh, Eiichiro Sando, Motoi Suzuki, Hiromi Fujita, Michio Yasunami, Keisuke Yoshihara, Lay-Myint Yoshida, Daniel Henry Paris, Koya Ariyoshi

**Affiliations:** 1 Department of Clinical Medicine, Institute of Tropical Medicine (NEKKEN), Nagasaki University, Nagasaki, Japan; 2 Nagasaki University Graduate School of Biomedical Sciences, Nagasaki, Japan; 3 Department of General Internal Medicine, Nagasaki Rosai Hospital, Nagasaki, Japan; 4 Department of Infectious Diseases, Bach Mai Hospital, Hanoi, Vietnam; 5 Department of General Internal Medicine, Fukushima Medical University, Fukushima, Japan; 6 The Partnership for Health Advancement in Vietnam (HAIVN), Hanoi, Vietnam; 7 Vietnam Research Station, Institute of Tropical Medicine (NEKKEN), Nagasaki University, Hanoi, Vietnam; 8 National Institute of Hygiene and Epidemiology, Hanoi, Vietnam; 9 Department of General Internal Medicine, Kameda Medical Center, Chiba, Japan; 10 School of Tropical Medicine and Global Health, Nagasaki University, Nagasaki, Japan; 11 Mahara Institute of Medical Acarology, Tokushima, Japan; 12 Saga-Ken Medical Centre Koseikan, Saga, Japan; 13 Department of Paediatric Infectious Diseases, Institute of Tropical Medicine (NEKKEN), Nagasaki University, Nagasaki, Japan; 14 Department of Medicine, Swiss Tropical and Public Health Institute, Basel, Switzerland; 15 Faculty of Medicine, University of Basel, Basel, Switzerland; Uniformed Services University of the Health Sciences, UNITED STATES

## Abstract

**Background:**

Scrub typhus (ST) is a leading cause of non-malarial febrile illness in Southeast Asia, but evidence of its true disease burden is limited because of difficulties of making the clinical diagnosis and lack of adequate diagnostic tests. To describe the epidemiology and clinical characteristics of ST, we conducted an observational study using multiple diagnostic assays at a national tertiary hospital in Hanoi, Vietnam.

**Methodology/Principal findings:**

We enrolled 1,127 patients hospitalized with documented fever between June 2012 and May 2013. Overall, 33 (2.9%) patients were diagnosed with ST by PCR and/or screening of ELISA for immunoglobulin M (IgM) with confirmatory tests: 14 (42.4%) were confirmed by indirect immunoperoxidase assay (IIP), and 19 (57.6%) were by IIP and PCR. Living by farming, conjunctival injection, eschar, aspartate aminotransferase elevation, and alanine aminotransferase elevation were significantly associated with ST cases (adjusted odds ratios (aORs): 2.8, 3.07, 48.8, 3.51, and 4.13, respectively), and having a comorbidity and neutrophilia were significantly less common in ST cases (aORs: 0.29 and 0.27, respectively). The majority of the ST cases were not clinically diagnosed with rickettsiosis (72.7%). Dominant IIP reactions against a single antigen were identified in 15 ST cases, whereas indistinguishably high reactions against multiple antigens were seen in 11 ST cases. The most frequently observed dominant IIP reaction was against Karp antigen (eight cases) followed by Gilliam (four cases). The highest diagnostic accuracy of IgM ELISA in acute samples was 78%. In a phylogenetic analysis of the 56-kDa type-specific antigen gene, the majority (14 cases) were located in the Karp-related branch followed by the Gilliam-related (two cases), Kato-related (two cases), and TA763-related clades (one case).

**Conclusions/Significance:**

Both the clinical and laboratory diagnoses of ST remain challenging at a tertiary hospital. Implementation of both serological and nucleic acid amplification assays covering endemic *O*. *tsutsugamushi* strains is essential.

## Author summary

Acute fever without localized signs is a common presentation of communicable neglected tropical diseases (NTDs). Scrub typhus, a potentially severe chigger-borne intracellular bacterial infection, is also an important differential diagnosis of acute febrile NTDs, which is endemic particularly in Asia. In Vietnam, the proportion of scrub typhus cases among patients with acute fever is currently unknown even in a tertiary hospital, because both clinical and laboratory diagnoses of scrub typhus are challenging. In this study, we enrolled acute fever patients hospitalized in the department of infectious diseases at a national tertiary hospital in Hanoi through a year. Scrub typhus was diagnosed using serological and PCR assays in 2.9% of enrolled patients. We found that the characteristics of scrub typhus patients were generally non-specific and most cases were missed based on clinical diagnosis alone. We also found that currently available serological assays were sub-optimal in Vietnam by comparing multiple diagnostic assays. Since the diagnostic antigens were not originally from Vietnam, the serological findings may underrepresent the actual number of cases. The presented data underlines the importance of a preemptive treatment strategy for scrub typhus among acute fever patients and adaptation of diagnostic assays to cover endemic strains.

## Introduction

Scrub typhus (ST) is caused by the obligate intracellular bacteria *Orientia tsutsugamushi*, which is transmitted by bites of trombiculid mites. Although scrub typhus is a leading cause of non-malarial febrile illness in Southeast Asia, evidence of the disease burden of scrub typhus is limited in some countries [1, 2]. In Vietnam, an acute fever study in the late 1960s showed that 11.6% of hospitalized American servicemen with acute fever were serologically diagnosed with ST [3]. Another observational study at a tertiary hospital in Hanoi, the capital city of Vietnam, in the early 2000s reported that 3.5% of admission cases were diagnosed as ST by an enzyme-linked immunosorbent assay (ELISA) [4]. Currently, there are no other published reports about the proportion of ST in undifferentiated fever patients in Vietnam.

One of the major reasons for the limited epidemiological evidence on ST is that making the clinical diagnosis is challenging. Similar to other rickettsioses, the initial presentation of ST is likely to be fever without localized signs. According to previous studies in Vietnam, the prevalence of specific clinical signs, such as eschar (46%-94%), rash (0%-34%), and lymphadenopathy (48.4%-85%), varies among study populations [5–8]. The broad variation and unreliability of clinical characteristics make physicians uncertain of clinically diagnosing ST.

In addition, the laboratory diagnosis of ST also poses a challenge [9]. Since the pathogen is an obligate intracellular bacterium, isolation requires specialized laboratory facilities and expertise. Serological tests that are considered to be the gold standard, such as indirect immunofluorescent assay (IFA) and indirect immunoperoxidase assay (IIP), should be performed using paired samples that are cumbersome to collect during routine clinical practice. Recently, immunoglobulin M (IgM) ELISA kits are emerging as an alternative to the gold standard serological tests [10]. Nevertheless, serological tests need to be adapted to the relevant endemic strains of *O*. *tsutsugamushi*, which means a batch of antigens is not always applicable everywhere. Both nested and/or real-time polymerase chain reaction (PCR) also serve as standard diagnostic methods. However, the sensitivity of PCR is not always sufficiently high, particularly for full blood samples [11]. Even today, depending assays used, multiple diagnostic assays are often required to confirm the diagnosis of ST.

In the present study, we aimed to improve our understanding on the epidemiology and characteristics of ST in Vietnam, and determine the proportion of ST cases among hospitalized patients with undifferentiated fever using multiple diagnostic assays. To investigate how to develop diagnostic assays appropriately for Vietnam, we also analyzed the genetic characteristics of local *O*. *tsutsugamushi* strains and the serological reaction patterns in ST cases.

## Methods

### Study design, participants, and enrollment criteria

We conducted a prospective observational study at the Department of Infectious Diseases of Bach Mai Hospital in Hanoi, Vietnam, which is the largest national referral hospital with approximately 1,900 beds. The hospital serves as referral center for all northern provinces of Northwest, Northeast, Red River Delta, and North Central Coast region of the country, where malaria is not endemic. We enrolled adult and adolescent patients who were admitted to the Department of Infectious Diseases between June 2012 and May 2013 based on the following eligibility criteria: 1) aged 13 years or older and 2) axillary temperature of ≥ 37.5°C at any time from disease onset to admission. We excluded patients with clinically definitive diagnoses on admission, such as mumps, mild cellulitis, food-borne gastroenteritis, rat-bite fever, dengue, and malaria (imported cases) based on apparent exposure or outbreak history within a group and specific signs and symptoms; patients with microbiologically confirmed diagnoses on admission (i.e., already diagnosed at a referral source); and patients with hepatitis-related diseases, because they were admitted to a specific ward for hepatic diseases. We assessed eligibility in daily morning meetings in the department on the next working day after admission.

### Study procedures and data collection

After obtaining informed consent, a trained research team member and local physician collected demographic and clinical information from medical charts to complete a standardized information sheet by the discharge day. The collected data included preceding antibiotic treatment, referral hospital name, and comorbidity. Clinical symptoms and physical signs were based on an initial assessment at admission. We extracted only the first results for repetitive laboratory tests. Clinical diagnosis, prescribed antibiotics, and patient outcomes were based on medical records up to the discharge date. All diagnostic and therapeutic plans during hospitalization were made by local physicians as routine clinical management.

Along with routine blood tests, 5 mL of blood was taken from all enrolled patients at the first blood draw that was stored in a tube with ethylenediaminetetraacetic acid (EDTA). The collected blood samples were centrifuged within 3 hours at 1010 x g (3000 revolutions per minute by CN-2060 (AS ONE Corporation, Osaka, Japan)) for 10 minutes, and each sample was divided into 5 tubes: one buffy-coat layer tube and 4 plasma tubes. All sample tubes were stored at -80°C until use. We took a second blood sample from patients hospitalized for more than three days along with clinical follow-up testing. The second blood samples were planned to be collected after seven hospital days. If a patient was to be discharged within seven days, a second blood sample was collected around the discharge day. The second blood samples were processed and stored the same way as the first samples were except that no buffy-coat sample was collected.

### Serological tests

Because we did not have capacity to test a large number of samples by standardized IFA or IIP, we adopted Scrub Typhus Detect IgM ELISA System (InBios International, Seattle, WA) for a serological screening test in the Institute of Tropical Medicine at Nagasaki University. Of the IgM ELISA-positive patients, the first and second paired samples were further tested by in-house IIP for IgM and IgG for confirmation at a referral laboratory (Mahara Institute of Medical Acarology, Tokushima, Japan).

#### IgM ELISA

To detect as many positive samples as possible, we screened all of the second set of plasma samples by IgM ELISA in accordance with the manufacturer’s instructions. In case of a patient had no second set of plasma collected, we screened a first set of plasma samples. We also tested the first set of plasma samples of patients whose second set of plasma screened positive. The results were read at 450 nm by Multiskan FC (Thermo Fisher Scientific, Waltham, MA). Based on the manufacturer’s recommendations and a previous report that used the same IgM ELISA kit in Thailand [10], we determined a cut-off optical density (OD) of 0.513, which was calculated by determining the mean OD value plus three standard deviations (SDs) of 68 control plasma samples collected from the visitors who received a general health examination at the Outpatient Department of Bach Mai Hospital.

#### IIP assay

The IIP was originally developed by Suto [12], and was improved by Fujita, who tested the study samples by previously reported methods [13–15]. The IIP was designed for diagnosing ST and similar endemic diseases in Japan using 9 different antigens, which were individually spotted on a slide glass, namely, *O*. *tsutsugamushi* serotypes Gilliam, Karp, Kato, Irie/Kawasaki, Hirano/Kuroki, Shimokoshi, *R*. *japonica*, *R*. *typhi*, and severe fever with thrombocytopenia syndrome (SFTS) virus. Since the IIP had not been previously tested in Vietnam, we defined the IIP positivity by a ≥ 4-fold increase in the IgM or IgG titer in the paired samples or a single IgM titer ≥ 320 (i.e., ≥ 1:320) with reference to diagnostic criteria in Japan [15].

### PCR and phylogenetic analysis

We extracted DNA from buffy coat samples using a QIAamp DNA Blood Mini Kit (QIAGEN, Hilden, Germany) in accordance with the manufacturer’s instructions. The DNA extracts were tested by a real-time PCR assay for the 47-kDa high-temperature requirement A protein (*HtrA*) gene using a QuantStudio 7 Flex Real-Time PCR System (Applied Biosystems, Waltham, MA) [16]. As confirmatory tests, we also tested DNA extracts of patients with real-time PCR- and/or IgM ELISA-positive results by two nested-PCR assays for the 47-kDa *HtrA* gene and 56-kDa type-specific antigen (*tsa*) gene using Veriti 200 thermal cycler (Applied Biosystems) [17, 18]. We analyzed the sequence of the nested-PCR products using an Applied Biosystems 3130xl Genetic Analyzer (Applied Biosystems). We also conducted phylogenetic analysis by MEGA6: Molecular Evolutionary Genetics Analysis Version 6.0 using the maximum likelihood method based on the Tamura-Nei model or the Hasegawa-Kishino-Yano model [19–21]. The all PCR assays and DNA sequence analyses were performed in the Institute of Tropical Medicine at Nagasaki University.

### Case definition

We defined ST cases as patients with positive results by more than one PCR assays or positive results by the IIP (a ≥ 4-fold increase in the IgM or IgG titer in the paired samples or a single IgM titer ≥ 320).

### Statistical analysis

Using an Access database (Microsoft Corporation, Redmond, WA), a trained research assistant entered data that were independently checked by three researchers (SK, SH, NCC) to correct for errors. We summarized categorical variables as frequencies and percentages, continuous variables as the mean or median and SD or inter-quartile range (IQR), respectively. Statistical comparisons between ST cases and non-ST patients were conducted by Fisher's exact test for categorical variables, the independent two-sample t-test for normally distributed continuous variables, and Wilcoxon rank-sum test for other continuous variables. We also calculated odds ratios with 95% confidence intervals by logistic regression analysis after multiple imputation to handle missing data. We imputed missing values using multivariate normal regression models including variables potentially correlated with being diagnosed as ST. We addressed continuous variables which were not normally distributed by log transformation in the imputation process. Further, to analyze diagnostic accuracy of the IgM ELISA and its cut-off OD value, we conducted receiver operating characteristic (ROC) analyses and estimated diagnostic parameters. We performed all analyses by STATA, version 13.1 (StataCorp LLC, College Station, TX). All tests were two-tailed and P < 0.05 was considered statistically significant. To identify a practical predictive model for diagnosing ST, we also conducted a classification and regression tree (CART) analysis using R [22].

### Ethics statement

Written informed consent was obtained from all adult participants and guardians of adolescent participants, and the data were analyzed anonymously. This study was approved by the institutional review boards and independent ethics committees of the Institute of Tropical Medicine at Nagasaki University (approval number: 12021085–4), Bach Mai Hospital and National Institute of Hygiene and Epidemiology as part of “Collaborative study on Emerging and Re-emerging infectious diseases in Vietnam” (approval number: 15-IRB, 2011).

## Results

### Investigation protocol

**Fig 1:**
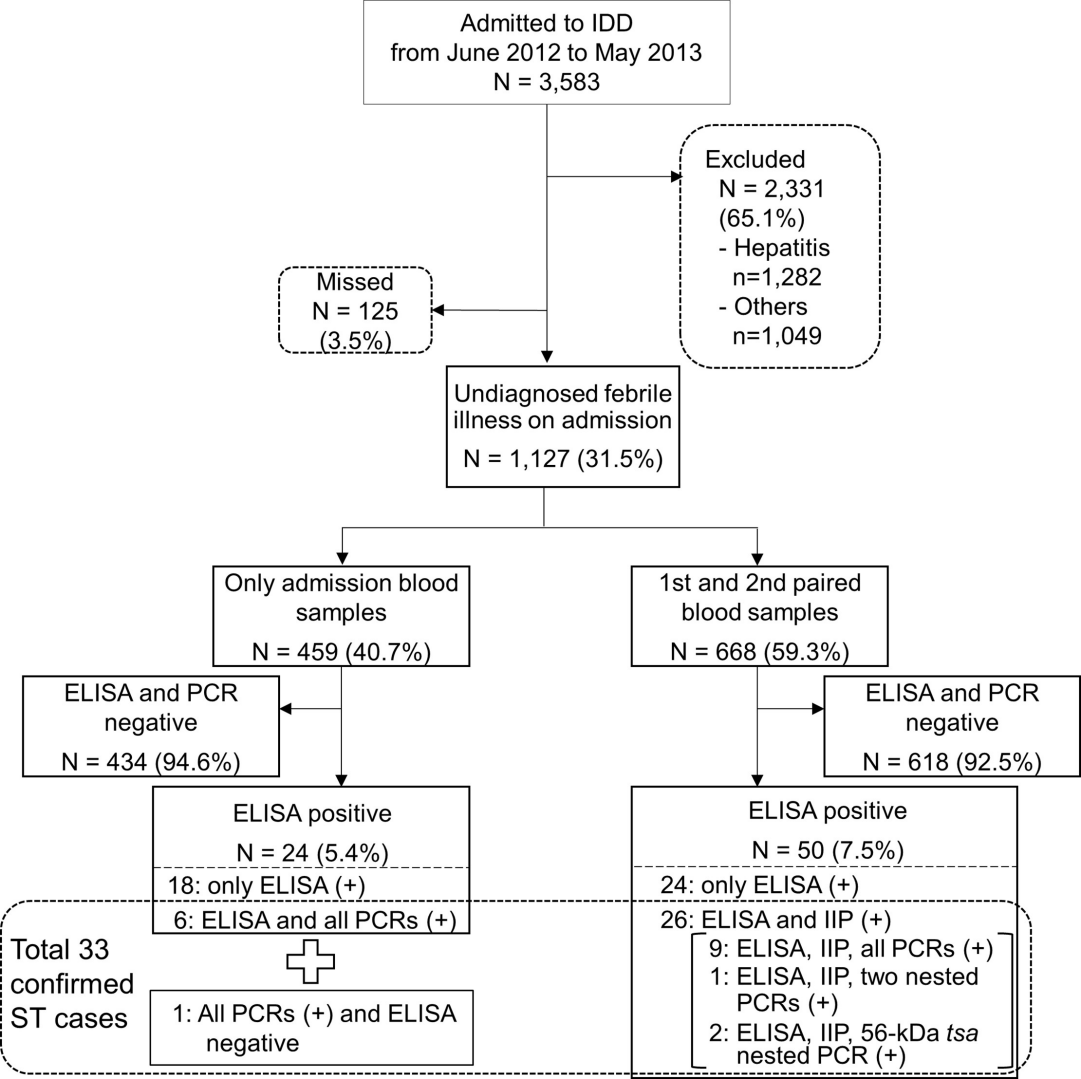
Investigation protocol for patients in the study. IDD: Department of Infectious Diseases, IIP: indirect immunoperoxidase assay, ST: scrub typhus, (+): positive test result.

The investigation protocol is summarized in Fig 1. A total of 3,583 patients admitted to the Department of Infectious Diseases of a full calendar year from June 2012 to May 2013 were screened. Of these, 2,331 were excluded due to having hepatitis-related diseases or known diagnoses on admission (S1 Fig); the eligibility of 125 patients was not assessed. In total, 1,127 patients with undiagnosed febrile illness on admission were enrolled to the study; paired blood samples were collected from 668, and only admission blood samples were collected from 459. The median (IQR) of days from fever onset to first blood collection were 8 (5–16, n = 1,112 with data available) and from fever onset to second blood collection were 14 (10–24, n = 665 with data available), respectively. Fifty of the 668 patients with paired blood samples were screened positive by ELISA, and further tested by IIP. Twenty-six of the 50 ELISA-positive patients with paired blood samples were diagnosed with ST by IIP. Among the patients with paired blood samples, nine cases were positive by real-time PCR and the two nested-PCRs. Of the 50 ELISA-positive patients, two cases were tested positive only by the nested-PCR for 56-kDa *tsa*, and one case was positive by the nested-PCRs for 47-kDa *HtrA* and 56-kDa *tsa*. All the PCR-positive cases were included in the 26 IIP-positive ST cases. Of the 459 patients with single available blood samples, 24 were tested positive by ELISA and seven were positive by real-time PCR. All the seven real-time PCR-positive patients were also positive by both nested PCRs to be diagnosed with ST. One of the seven ST cases was negative by ELISA and the other six were positive. Other ELISA-positive patients were negative by PCR, and IIP was not performed for patients with single available blood samples. Overall, 33 patients were confirmed as ST cases.

### Demographic and clinical characteristics

The demographic and clinical characteristics of the participants are compared in Table 1. There were significant differences between the two groups. The proportions of farmers (60.6%) and residents outside of Hanoi (84.9%) were higher in ST cases, whereas comorbidity (9.1%) was lower. Nearly half of each group was referred from another hospital. Headache (66.7%) was commonly observed, and no diarrhea was observed in ST cases. The proportion of tachycardiac patients was lower in ST cases (6.9%). Although conjunctival injection and eschar were more frequent in ST cases, the frequencies were not extremely high (39.4%, and 18.2%, respectively). Rash, hepatomegaly or splenomegaly, and multiple lymphadenopathies did not occur significantly more in ST cases than in non-ST cases. Severely ill patients who underwent a quick sequential organ failure assessment (qSOFA) scored higher than one were uncommon in both groups (< 10%). The proportion of neutrophilia was lower in ST cases (18.2%), whereas elevated aspartate aminotransferase (AST) and alanine aminotransferase (ALT) were significantly more frequent in ST cases (71.9% each). Unexpectedly, only a small proportion of the laboratory-diagnosed ST cases was diagnosed with rickettsiosis at discharge (27.3%), while 14 patients who were diagnosed with rickettsiosis at discharge did not receive a laboratory diagnosis of ST. Discharge diagnosis categories of all participants are summarized in S1 Table. As an in-hospital treatment, 69.7% of ST cases were treated with effective antibiotics such as doxycycline or azithromycin. Complete recovery at discharge was more frequently achieved in ST cases (57.6%).

**Table 1:** Comparison of the demographic and clinical characteristics.

	ST cases N = 33 n (%)	Non-ST cases N = 1094 n (%)	P value*
**Basic information**			
Age, years (mean, SD) (N = 1127)	45.0, 15.9	44.8, 17.7	0.945
Female sex	18 (54.5)	495 (45.2)	0.294
Farmer	20 (60.6)	358 (32.7)	**0.001**
Living outside of Hanoi	28 (84.9)	730 (66.7)	**0.037**
Admission in the rainy season (May-October)	20 (60.6)	608 (55.6)	0.599
Preceding antibiotic treatment (N = 393)	3 (21.4) (N = 14)	116 (30.6) (N = 379)	0.566
Referral from another hospital	17 (51.5)	516 (47.2)	0.724
Having comorbidity	3 (9.1)	291 (26.6)	**0.025**
**Symptoms**			
Fever duration before admission, days (median, IQR) (N = 1112)	7, 5–10	7, 3–15 (N = 1079)	0.512
Rigor	8 (24.2)	354 (32.4)	0.449
Headache	22 (66.7)	509 (46.5)	**0.032**
Sore throat	2 (6.1)	80 (7.3)	1
Cough	14 (42.4)	380 (34.7)	0.36
Sputum	4 (12.1)	202 (18.5)	0.493
Abdominal pain	7 (21.2)	200 (18.3)	0.649
Nausea/vomiting	7 (21.2)	249 (22.8)	1
Diarrhea	0 (0.0)	142 (13.0)	**0.016**
Myalgia	7 (21.2)	150 (13.7)	0.207
Arthralgia	0 (0.0)	97 (8.9)	0.106
**Physical signs**			
Axillary temperature > 38°C (N = 1106)	16 (48.5)	424 (39.5) (N = 1073)	0.367
Heart rate > 90 beats/min (N = 1089)	2 (6.9) (N = 29)	256 (24.2) (N = 1060)	**0.027**
Systolic blood pressure ≤ 100 mmHg (N = 1121)	4 (12.1)	205 (18.8) (N = 1088)	0.495
qSOFA score** ≥ 2 (N = 892)	1 (4.6) (N = 22)	77 (8.9) (N = 870)	0.713
Injected conjunctiva	13 (39.4)	182 (16.6)	**0.002**
Pharyngeal injection	9 (27.3)	207 (18.9)	0.259
Lung rales	8 (24.2)	277 (25.3)	1
Abdominal tenderness	3 (9.1)	100 (9.1)	1
Hepatomegaly and/or splenomegaly	8 (24.2)	205 (18.7)	0.496
Rash	3 (9.1)	136 (12.4)	0.789
Eschar	6 (18.2)	8 (0.7)	**< 0.001**
Multiple lymphadenopathies	4 (12.1)	49 (4.5)	0.065
Neck stiffness	2 (6.1)	98 (9.0)	0.762
**Laboratory**			
Days from fever onset to 1st blood collection (median, IQR) (N = 1112)	8, 7–10	8, 5–16 (N = 1079)	0.558
Days from fever onset to 2nd blood collection (median, IQR) (N = 665)	13.5, 12–16 (N = 26)	14, 10–25 (N = 639)	0.705
Hemoglobin < 10.0 g/dL (N = 1123)	4 (12.1)	188 (17.3) (N = 1090)	0.638
Platelet < 150,000 /μL (N = 1114)	12 (36.4)	257 (23.8) (N = 1081)	0.101
WBC > 12,000 /μL (N = 1123)	7 (21.2)	396 (36.3) (N = 1090)	0.096
Absolute neutrophil count > 8,000 /μL (N = 1105)	6 (18.2)	468 (43.7) (N = 1072)	**0.004**
AST > 40 IU/L (N = 1087)	23 (71.9) (N = 32)	444 (42.1) (N = 1055)	**0.001**
ALT > 40 IU/L (N = 1079)	23 (71.9) (N = 32)	387 (37.0) (N = 1047)	**< 0.001**
Creatinine > 1.3 mg/dL (N = 1082)	3 (9.4) (N = 32)	137 (13.1) (N = 1050)	0.789
CRP > 10 mg/dL (N = 1007)	7 (21.9) (N = 32)	313 (32.1) (N = 975)	0.252
**Clinical diagnosis, treatment & outcome**			
Clinical diagnosis of rickettsiosis	9 (27.3)	14 (1.3)	**< 0.001**
Treated by DOXY/AZM	23 (69.7)	437 (40.0)	**0.001**
Complete recovery at discharge	19 (57.6)	325 (29.7)	**0.002**
Prolonged fever after 5th hospital day (N = 992)	8 (25.0) (N = 32)	148 (15.4) (N = 960)	0.142

* P values were calculated by Fisher's exact test for categorical variables, the independent two-sample t-test for normally distributed continuous variables, and Wilcoxon rank-sum test for other continuous variables.

** qSOFA score: quick sequential organ failure assessment score (range 0–3 points, with 1 point each for systolic hypotension [≤ 100mmHg], tachypnea [≥ 22 /min], or altered mental status [Glasgow coma scale < 15]).

ST: scrub typhus, ELISA (+): positive results by enzyme-linked immunosorbent assay, PCR (-): negative results by polymerase chain reaction, SD: standard deviation, IQR: interquartile range, WBC: white blood cell, AST: aspartate transaminase, ALT: alanine transaminase, CRP: C-reaztive protein, DOXY/AZM: doxycycline or azithromycin.

Crude and adjusted odds ratios of being diagnosed as ST by adjusting for age > 50, gender, living by farming, living outside of Hanoi, and having a comorbidity with missing data imputed by multiple imputation except for preceding antibiotic treatment and days from fever onset to the second blood collection are shown in Table 2. Although the statistical significance of adjusted odds ratios disappeared in living outside of Hanoi, headache, diarrhea, and heart rate > 90 beats per minute, other significantly different characteristics remained significant after adjustment. Using the significant variables and potentially correlated variables (age, sex, living by farming, living outside of Hanoi, having a comorbidity, headache, diarrhea, heart rate, conjunctival injection, eschar, multiple lymphadenopathies, neutrophil count, platelet count, AST, and ALT), we conducted a CART analysis to make a diagnostic prediction model in the 1,127 participants. Although the model was able to correctly differentiate ST cases in 17 out of 33 (51.5%) cases, predictive value of ST was fallen within 43–47% by conditions of eschar, AST, farming, heart rate, and neutrophil count (S2 Fig).

**Table 2:** Crude and adjusted odds ratios of being diagnosed as ST by demographic and clinical characteristics.

	cOR (95% CI)	P value*	aOR** (95% CI)	P value*
**Basic information**				
Age > 50	0.91 (0.44–1.87)	0.797	0.93 (0.45–1.93)	0.847
Female sex	1.45 (0.72–2.91)	0.293	1.36 (0.67–2.76)	0.392
Farmer	3.16 (1.56–6.43)	**0.001**	2.8 (1.36–5.74)	**0.005**
Living outside of Hanoi	2.79 (1.07–7.29)	**0.036**	2.45 (0.92–6.49)	0.072
Admission in the rainy season (May-October)	1.23 (0.61–2.50)	0.567	1.1 (0.53–2.25)	0.799
Preceding antibiotic treatment (N = 393)	0.62 (0.17–2.26)	0.467	0.65 (0.18–2.44)	0.526
Referral from another hospital	1.19 (0.60–2.38)	0.622	1.14 (0.56–2.32)	0.725
Having a comorbidity	0.28 (0.08–0.91)	**0.035**	0.29 (0.09–0.95)	**0.042**
**Symptoms**				
Fever duration before admission > 7 days (N = 1112)^#^	0.71 (0.34–1.48)	0.358	0.63 (0.30–1.34)	0.228
Rigor	0.67 (0.30–1.50)	0.328	0.56 (0.25–1.28)	0.171
Headache	2.3 (1.10–4.79)	**0.026**	2.11 (1.00–4.46)	0.051
Sore throat	0.82 (0.19–3.48)	0.785	0.95 (0.22–4.15)	0.95
Cough	1.38 (0.69–2.79)	0.363	1.49 (0.73–3.06)	0.271
Sputum	0.61 (0.21–1.75)	0.358	0.66 (0.23–1.93)	0.448
Abdominal pain	1.2 (0.51–2.81)	0.669	1.14 (0.48–2.71)	0.761
Nausea/vomiting	0.91 (0.39–2.13)	0.834	0.82 (0.35–1.94)	0.655
Diarrhea	NA (NA)	NA	NA (NA)	NA
Myalgia	1.69 (0.72–3.97)	0.225	1.57 (0.66–3.75)	0.306
Arthralgia	NA (NA)	NA	NA (NA)	NA
**Physical signs**				
Axillary temperature > 38°C (N = 1106)^#^	1.43 (0.71–2.86)	0.313	1.48 (0.73–2.98)	0.277
Heart rate > 90 beats/min (N = 1089)^#^	0.31 (0.08–1.20)	0.089	0.30 (0.08–1.18)	0.085
Systolic blood pressure ≤ 100 mmHg (N = 1121)^#^	0.59 (0.21–1.71)	0.335	0.51 (0.17–1.49)	0.217
qSOFA score^$^ ≥ 2 (N = 892)^#^	0.77 (0.19–3.16)	0.714	0.73 (0.17–3.05)	0.664
Injected conjunctiva	3.26 (1.59–6.67)	**0.001**	3.07 (1.46–6.43)	**0.003**
Pharyngeal injection	1.61 (0.74–3.51)	0.234	1.77 (0.79–3.94)	0.164
Lung rales	0.94 (0.42–2.12)	0.888	1.13 (0.49–2.61)	0.771
Abdominal tenderness	0.99 (0.30–3.32)	0.992	0.96 (0.28–3.27)	0.953
Hepatomegaly and/or splenomegaly	1.39 (0.62–3.12)	0.428	1.49 (0.65–3.42)	0.347
Rash	0.7 (0.21–2.34)	0.567	0.72 (0.21–2.41)	0.59
Eschar	30.2 (9.79–94.95)	**< 0.001**	48.8 (13.02–182.93)	**< 0.001**
Multiple lymphadenopathies	2.94 (1.00–8.70)	0.051	2.7 (0.88–8.32)	0.083
Neck stiffness	0.66 (0.15–2.78)	0.567	0.59 (0.14–2.55)	0.483
**Laboratory**				
Days from fever onset to 1st blood collection ≤ 7 days (N = 1112)^#^	0.58 (0.28–1.22)	0.151	0.61 (0.29–1.28)	0.191
Days from fever onset to 2nd blood collection > 21 (N = 665)	0.46 (0.16–1.35)	0.159	0.4 (0.13–1.21)	0.104
Hemoglobin < 10.0 g/dL (N = 1123)^#^	0.66 (0.23–1.90)	0.444	0.7 (0.24–2.05)	0.513
Platelet < 150,000 /μL (N = 1114)^#^	1.82 (0.88–3.75)	0.104	2.00 (0.96–4.18)	0.065
WBC > 12,000 /μL (N = 1123)^#^	0.47 (0.20–1.10)	0.081	0.45 (0.19–1.06)	0.067
Absolute neutrophil count > 8,000 /μL (N = 1105)^#^	0.28 (0.12–0.69)	**0.006**	0.27 (0.11–0.67)	**0.004**
AST > 40 IU/L (N = 1087)^#^	3.46 (1.59–7.54)	**0.002**	3.51 (1.59–7.75)	**0.002**
ALT > 40 IU/L (N = 1079)^#^	4.29 (1.97–9.35)	**< 0.001**	4.13 (1.87–9.10)	**< 0.001**
Creatinine > 1.3 mg/dL (N = 1082)^#^	0.72 (0.22–2.33)	0.579	0.87 (0.26–2.95)	0.829
CRP > 10 mg/dL (N = 1007)^#^	0.64 (0.27–1.48)	0.294	0.68 (0.29–1.62)	0.385
**Clinical diagnosis, treatment & outcome**				
Clinical diagnosis of rickettsiosis	28.93 (11.41–73.32)	**< 0.001**	22.34 (8.27–60.38)	**< 0.001**
Treated by DOXY/AZM	3.46 (1.63–7.34)	**0.001**	3.22 (1.50–6.91)	**0.003**
Complete recovery at discharge	3.21 (1.59–6.48)	**0.001**	2.84 (1.38–5.85)	**0.005**
Prolonged fever after 5th hospital day (N = 992) ^#^	1.36 (0.60–3.09)	0.457	1.42 (0.61–3.29)	0.415

* P values were calculated by univariable logistic regression analysis for crude odds ratios and multivariable logistic regression analysis for adjusted odds ratios.

** Adjusted for age > 50, gender, living by farming, living outside of Hanoi, and having a comorbidity.

# Missing values were imputed by multiple imputation using multivariate normal regression models including all the variables with missing values, age, gender, living by farming, living outside of Hanoi, having a comorbidity, and ST diagnosis.

$ qSOFA score: quick sequential organ failure assessment score (range 0–3 points, with 1 point each for systolic hypotension [≤ 100 mmHg], tachypnea [≥ 22 /min], or altered mental status [Glasgow coma scale < 15]).

ST: scrub typhus, cOR: crude odds ratio, aOR: adjusted odds ratio, CI: confidence interval, NA: not available, WBC: white blood cell, AST: aspartate transaminase, ALT: alanine transaminase, CRP: C-reactive protein, DOXY/AZM: doxycycline or azithromycin.

### Serological assessment

The 50 patients who were screened positive by IgM ELISA with paired plasma samples were tested by IIP. Summary of the 50 patients’ diagnostic test results and the individual results are shown in Table 3 and S2 Table, respectively. The median (IQR) of days from fever onset to the first blood collection and the interval between the first and second blood collection of the 50 patients were 8 (7–13) and 5 (4–6), respectively. Of the 50 patients, 26 were diagnosed with ST, and dominant IIP reaction against a single antigen was identified in 15 ST cases, whereas indistinguishably high reactions against multiple antigens were seen in 11 ST cases. The most frequently observed dominant IIP reaction was against Karp antigen (eight cases) followed by Gilliam (four cases), Hirano/Kuroki (one case), Irie/Kawasaki (one case), Shimokoshi (one case). Indistinguishably high IIP reactions were seen most frequently against Karp/Kato (four cases), followed by against Karp/Kato/Hirano (two cases). Interestingly, there was a case (ID: 91) with apparent IgM seroconversion against the Shimokoshi antigen, whose ELISA OD value was relatively low (S2 Table). Among the 50 paired blood samples, the median (IQR) OD values of IgM ELISA in the first and second blood samples were 0.534 (0.384–1.748) and 1.070 (0.623–3.217), respectively. Summary of ROC analysis and ROC curve of ST diagnosis by IgM ELISA in the first blood samples are shown in Table 3 and S3 Fig. When we set the cut-off OD value at 0.513 for screening in the first blood samples, diagnostic accuracy, sensitivity, and specificity were 68%, 69.2%, and 66.7%, respectively. When we set the cut-off OD value at 1.576 in the first blood samples, where we observed the highest accuracy; diagnostic accuracy, sensitivity, and specificity were 78%, 57.7%, and 100%, respectively. Similarly, we observed the highest accuracy by the cut-off OD value at 1.599 in the second blood samples (Table 3, S4 Fig), and the diagnostic accuracy, sensitivity, and specificity were 84%, 73.1%, and 95.8%, respectively.

**Table 3:** Summary of diagnostic test results in the 50 patients screened positive by the second blood-IgM ELISA.

Days from fever onset to the 1st blood collection (median, IQR)	8, 7–13
Days from fever onset to the 2nd blood collection (median, IQR)	14, 11–20
Days of interval between the 1st and 2nd blood collection (median, IQR)	5, 4–6
Number of patients with ST diagnosis	26
IgM or IgG titer ≥ 4-fold increase by IIP (with positive by any PCR)	14 (4)
Single IgM titer ≥ 320 (i.e., ≥ 1:320) by IIP (with positive by any PCR)	12 (8)
Positive by more than one PCR assays* (with single IgM titer ≥ 320 by IIP)	10 (6)
Dominant IIP reactions against a single antigen	15
against Karp antigen	8
against Kato antigen	0
against Gilliam antigen	4
against Hirano/Kuroki antigen	1
against Irie/Kawasaki antigen	1
against Shimokoshi antigen	1
Indistinguishable IIP reactions against multiple antigens	11
IgM ELISA OD value of the 1st blood sample (median, IQR)	0.534, 0.384–1.748
IgM ELISA OD value of the 2nd blood sample (median, IQR)	1.070, 0.623–3.217
AUC of ROC curve of ST diagnosis by IgM ELISA in the 1st blood samples (95% CI)	0.73 (0.57–0.89)
Diagnostic parameters by cut-off OD value at 0.513 in the 1st blood sample-IgM ELISA	
accuracy (95% CI)	68% (53.3%-80.5%)
sensitivity (95% CI)	69.2% (48.2%-85.7%)
specificity (95% CI)	66.7% (44.7%-84.4%)
positive predictive value (95% CI)	69.2% (48.2%-85.7%)
negative predictive value (95% CI)	66.7% (44.7%-84.4%)
Diagnostic parameters by cut-off OD value at 1.576 in the 1st blood sample-IgM ELISA^#^	
accuracy (95% CI)	78% (64.0%-88.5%)
sensitivity (95% CI)	57.7% (36.9%-76.6%)
specificity (95% CI)	100% (85.8%-100%)
positive predictive value (95% CI)	100% (78.2%-100%)
negative predictive value (95% CI)	68.6% (50.7%-83.1%)
AUC of ROC curve of ST diagnosis by IgM ELISA in the 2nd blood samples (95% CI)	0.91 (0.83–0.99)
Diagnostic parameters by cut-off OD value at 1.599 in the 2nd blood sample-IgM ELISA^#^	
accuracy (95% CI)	84% (70.9%-92.8%)
sensitivity (95% CI)	73.1% (52.2%-88.4%)
specificity (95% CI)	95.8% (78.9%-99.9%)
positive predictive value (95% CI)	95% (75.1%-99.9%)
negative predictive value (95% CI)	76.7% (57.7%-90.1%)

* All the PCR-positive patients also fulfilled the diagnostic criteria by IIP.

# The presented cut-off OD values at 1.576 and 1.599 produced the highest accuracy in the ROC analyses.

IQR: interquartile range, ST: scrub typhus, IIP: indirect immunoperoxidase assay, OD: optical density, AUC: area under the curve, ROC: receiver operating characteristic, CI: confidence interval

### Phylogenetic analysis and case distribution

**Fig 2:**
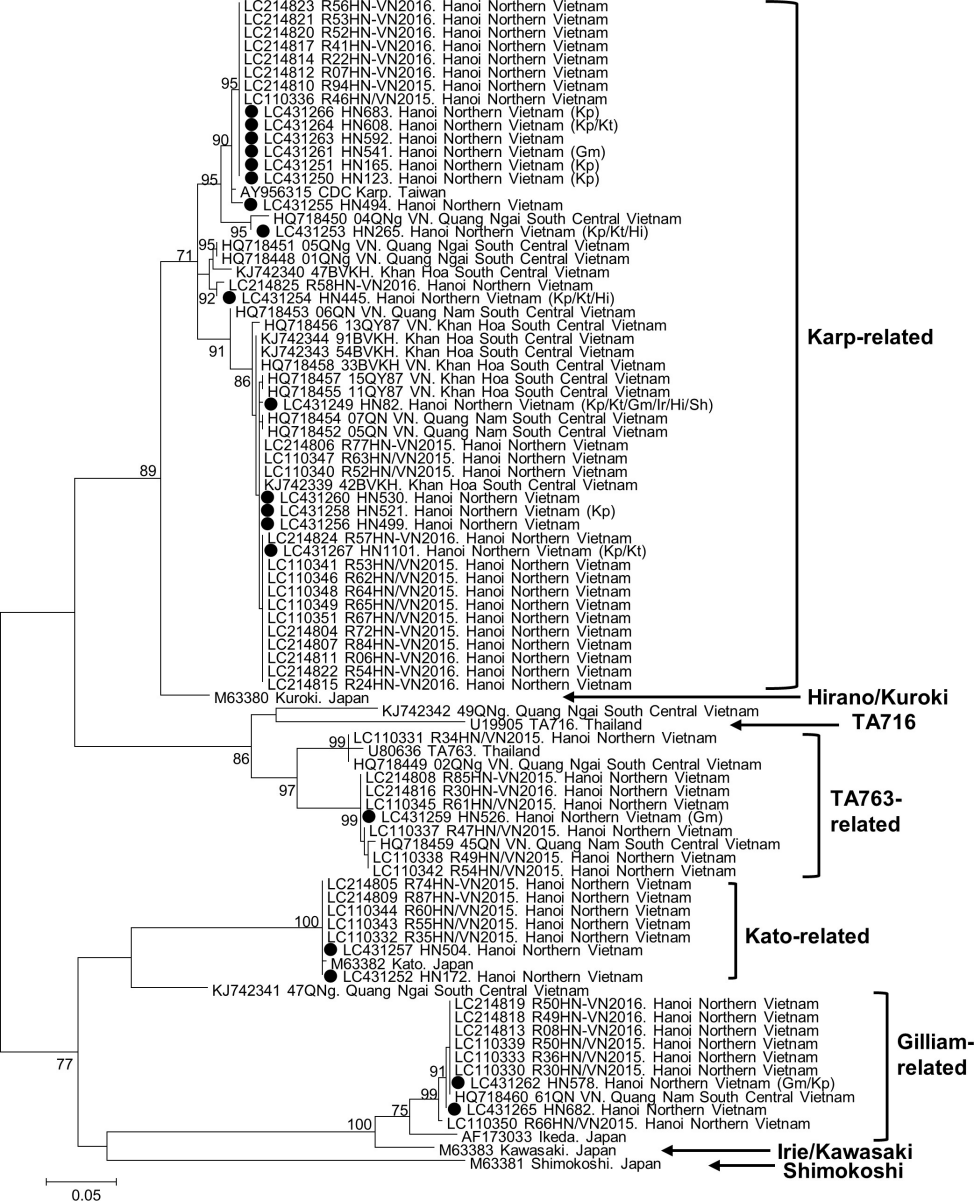
Phylogenetic tree of *O*. *tsutsugamushi* 56-kDa *tsa* gene sequences of the strains reported in Vietnam. GenBank accession number, strain name, and reported place are indicated. The evolutionary history was inferred by using the maximum likelihood method based on the Tamura-Nei model with a discrete gamma distribution with a bootstrap test of 1000 replicates. Bootstrap values higher than 70 were considered to be statistically significant and are shown next to the branches. The tree is drawn to scale, with branch lengths measured using the number of substitutions per site. All positions with less than 95% site coverage were eliminated. That is, fewer than 5% of alignment gaps, missing data, and ambiguous bases were allowed at any position. There were a total of 393 positions in the final dataset. Evolutionary analyses were conducted with MEGA6. Types of antigen with the highest reaction by immunoperoxidase assay are indicated with a bracket, if the patient was tested. Kp: Karp, Kt: Kato, Gm: Gilliam, Hi: Hirano/Kuroki, black circle: strains in the present study (GenBank accession numbers: LC431249-LC431267).

**Fig 3:**
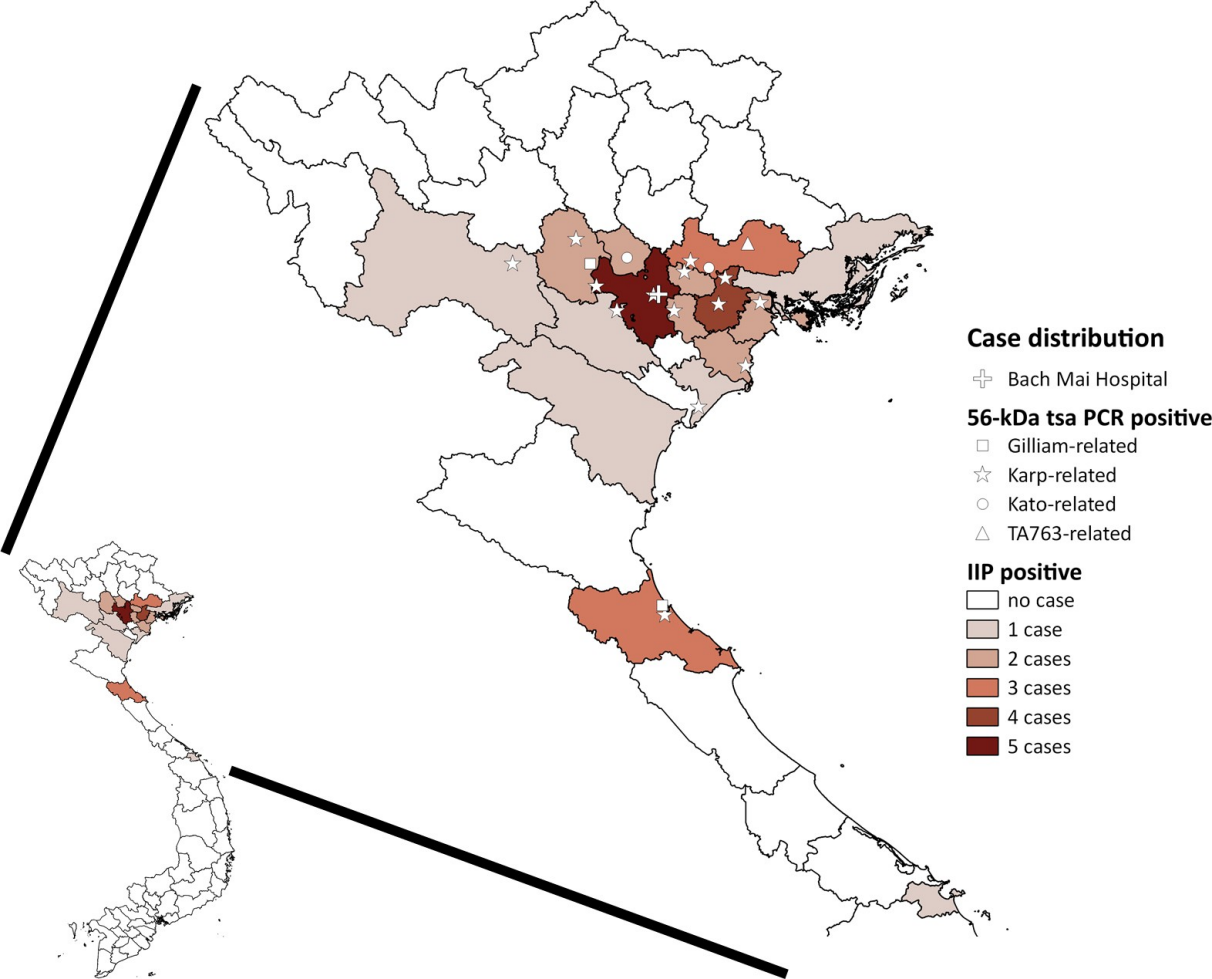
Geographical distribution of patients’ home addresses with positive results by 56-kDa *tsa* PCR and/or IgM ELISA, and the location of Bach Mai Hospital. The numbers of IgM ELISA-positive cases were counted by province. The map image was created by QGIS 2.18.11 software (Open Source Geospatial Foundation Project. https://www.qgis.org) using cartographic data files extracted from the GADM database (www.gadm.org), version 2.5 (July 2015). IIP: indirect immunoperoxidase assay.

We analyzed the sequences of all of the nested PCR products of the 56-kDa *tsa* and 47-kDa *HtrA* genes (N = 19 and 17, respectively). One sample (ID: 526) had double peaks of equivalent heights at eight loci of the 47-kDa *HtrA* gene chromatogram but no double peaks in the 56-kDa *tsa* gene, indicating a case of multistrain infection. A phylogenetic tree of 393 base pairs of the 56-kDa *tsa* gene fragment is shown in Fig 2, which includes previously reported strains in Vietnam and the strains from the present study (GenBank accession numbers: LC431249-LC431267). The majority (N = 14) were located at the Karp-related branch, followed by the Gilliam-related (N = 2), Kato-related (N = 2), and TA763-related (N = 1) clades. IIP reaction data were not available in seven cases (four Karp-related, two Kato-related and one Gilliam-related). Of 12 cases with both genotype and IIP results, the 56-kDa *tsa* gene genotype was compatible with the antigen with either dominant or one of indistinguishably high IIP reaction in 10 cases (nine Karp-related and one Gilliam-related), whereas the genotype was incompatible with IIP results in two cases with dominant IIP reaction against Gilliam, of which one had TA763 genotype and the other one had Karp-related genotype (ID: 526 and 541, respectively) (S2 Table). A phylogenetic tree of 657 base pairs of the 47-kDa *HtrA* gene fragment was also generated (S5 Fig), which showed less diversity than the 56-kDa *tsa* gene-based tree (GenBank accession numbers: LC431268-LC431284). The geographical distribution of the addresses of all of the ELISA-positive cases and the 56-kDa *tsa* genotypes are described in Fig 3. Most patients lived in Hanoi and nearby provinces, whereas the ST cases were distributed throughout northern Vietnam. Although the number of samples was minimal, no significant genotype cluster is seen on the map.

## Discussion

This study describes the demographic and clinical characteristics of 33 (2.9%) cases diagnosed with ST by IgM ELISA, IIP, and/or PCR assays out of 1,127 hospitalized patients with undifferentiated fever. It should be noted that the majority of the ST cases were not clinically diagnosed primarily with rickettsiosis; only 27.3% of ST cases were diagnosed with rickettsiosis at discharge. This was probably because many of the ST cases did not present with characteristic clinical signs and symptoms. The results of CART analysis showed that 48.5% of ST cases were unpredictable by a diagnostic model using relatively characteristic clinical information. These findings illustrate how challenging it is to diagnose ST at a tertiary hospital in an endemic country.

However, as seen in previous studies [5–8], the clinical characteristics of ST cases can vary depending on the study population, even in a study conducted at the same site. Our previous study that was conducted at Bach Mai Hospital diagnosed 237 (41.0%) ST cases by an IgM ELISA kit out of 579 patients with suspected rickettsiosis between 2001 and 2003 [5]. When we applied the same criteria of suspected rickettsiosis, the present study diagnosed only 16 (4.0%) cases out of 402 patients with suspected rickettsiosis. We believe that this clear difference in the ST prevalence between the previous and present studies can be explained by different case definitions; the previous study depended solely on IgM ELISA but the present study used IgM ELISA for screening, and IIP and PCRs for confirmation. In addition, Bach Mai Hospital functions as an educational medical center, has published local guidelines for internal medicine including rickettsiosis in 2013, and has provided clinical and diagnostic training for local physicians [23]. Consequently, typical ST cases were likely to have been diagnosed and treated at a community hospital level and were less frequently referred in recent years. The high proportion of ST cases localizing to the outskirts in this study (Fig 3), suggests that ST is a more rural distributed disease, as was previously described in other countries in Southeast Asia [24]. However, recent economic development of the country may have reduced the risk of ST by the shift in workforce from agriculture to urban occupation, rapid urbanization and loss of wilderness, and the changes in farming practices and equipment. To correctly assess the disease burden of ST, primary and secondary care facilities, where more cases of ST may be clinically diagnosed and treated, should be involved. Therefore, the introduction of a national surveillance system is necessary.

We identified 75 patients screened positive by IgM ELISA using a diagnostic cut-off OD value at 0.513, which is estimated by the manufacturer’s recommended methods. However, the IgM ELISA cut-off OD value was based on the plasma samples from general health check visitors at the reference hospital in Hanoi. Although Bach Mai Hospital accepts general health check visitors from all over the provinces in northern Vietnam, it is unclear whether our control samples could represent rural populations, because the individual information of the control group including address was not available. When we compared the 42 non-ST patients with IgM ELISA-positive results to 33 ST cases and 1,052 ELISA-negative patients, the clinical characteristics were different from ST cases and relatively less different from ELISA-negative patients (S3 Table). This indicates that the employed cut-off OD value 0.513 was likely to have caused a substantial number of false positives. As a result, the diagnostic accuracy, sensitivity, and specificity of IgM ELISA with a cut-off OD value at 0.513 in the acute phase samples were all less than 70%. The higher cut-off OD value 1.576 in the acute phase samples led to the higher specificity and even lower sensitivity. Although slightly higher sensitivity was seen by cut-off OD value at 1.599 in the second blood samples, it was at most 73.1% and cannot contribute early diagnosis in clinical setting. In previous diagnostic accuracy studies assessing the same IgM ELISA kit in Thailand, India, and Bangladesh, the optimal cut-off OD values varied considerably from 0.2–0.6, 0.41–1.0, to 0.75–1.25, respectively [10, 25, 26]. The optimum cut-off OD values depends on the background antibody titers of the study populations. Because our study was a single tertiary medical center-based study, encompassing a wide range of northern Vietnam populations, it is even more complex to determine the optimal cut-off OD value.

The IgM ELISA kit uses a mixed antigen of recombinant p56-kDa Karp, Kato, Gilliam, and TA716 strain antigens, while the IIP uses six discrete whole-cell cultured antigens of the Karp, Kato, Gilliam, Irie/Kawasaki, Hirano/Kuroki, and Shimokoshi strains, which are all endemic in Japan. Although the current IIP has not been evaluated in northern Vietnam, the results indicate some possibilities that should be addressed. We observed various degrees of discrepancy between the IIP reactions and ELISA. Some cases, such as ID 380, 822 and 1062, showed no IIP reaction for both IgM and IgG despite having an OD value of > 0.6 (S2 Table). Other cases, such as ID 91 and ID 258, had dominant IIP reactions against the Shimokoshi or Irie/Kawasaki strains and showed a relatively low OD value (S2 Table). These cases might have been infected with unknown endemic strains of *O*. *tsutsugamushi*, which do not react well with the Japanese strains or with the strains used by the IgM ELISA kit. Without doubt, neither the antigen combination of endemic pathogens in Japan nor the IgM ELISA is optimal for diagnosing ST in northern Vietnam, and the isolation and characterization of endemic strains is a necessary task. Therefore, these hypotheses should be tested in future studies by comparing the IgM ELISA kit with a gold standard serological assay using an optimized combination of discrete whole-cell antigens including locally isolated strains such as IIP or indirect immunofluorescent assay.

The phylogenetic analysis described genetic diversity in 56-kDa *tsa* in northern Vietnam, which is similar to the previous studies in Hanoi and South Central Coast region [6, 27, 28]. The results show that genetic variety of the 56-kDa *tsa* is common in Vietnam, although the Karp-related type is apparently dominant. As previously reported [17], the 47-kDa *HtrA* gene is more conserved than the 56-kDa *tsa* gene in our results. However, both the 56-kDa *tsa* and 47-kDa *HtrA* gene types were discordant with the serotype by IIP in several cases. This may be because of antigens other than 56-kDa/47-kDa of whole-cell cultured *O*. *tsutsugamushi* that are used by the IIP, or difference between the antigens used by the IIP and endemic strains. This is further evidence documenting that genotype and serotype are not the same. Although 56-kDa *tsa* is known as a major serological antigen against *O*. *tsutsugamushi* [29], serological antigenicity might be better described by whole genome or multi-locus sequence analysis than by single gene sequence analysis.

The present study has several limitations. In addition to that there is no evidence about the validity of the IgM ELISA and IIP assay and their cut-off values in Vietnam, there is no evidence about endemic strains based on serotype in the study setting. The IIP covers classic standard serotypes Gilliam, Karp, and Kato, with additional three Japanese serotypes Irie/Kawasaki, Hirano/Kuroki, and Shimokoshi; which could lead to a high probability to observe serological reaction (some could be cross-reaction) caused by local strains. However, there is no doubt that a batch of antigens used in serological tests should include local ST strains, otherwise serotyping remain difficult as shown in this paper. It is important to be aware that the present study aimed to describe the ST cases encountered at a tertiary hospital in northern Vietnam; thus, the present findings cannot be generalized to the primary care level. It is necessary to conduct further studies at the primary and secondary health care level to comprehensively determine the ST prevalence in northern Vietnam. In addition, the presented results might underrepresent the actual ST prevalence, as clinically diagnosed dengue and febrile hepatitis cases were not included, which could have masked additional ST cases. We could not apply all the diagnostic tests to all collected samples, which particularly weaken the evidence of diagnostic parameters. Furthermore, the selection of the cut-off values of the IgM ELISA and IIP could have impacted on the results of diagnostic parameters. However, we believe that the present study clearly demonstrates the difficulties faced by physicians in tertiary clinical settings in northern Vietnam in clinically distinguishing ST cases from many other febrile diseases. The proportion of wrongly or under treated patients also reflect that in areas where ST is common adequate antibiotics should be considered in pre-emptive treatment strategies, until diagnosis is confirmed. To overcome this diagnostic challenge, implementation of both serological assays and PCRs covering endemic *O*. *tsutsugamushi* strains is essential, which means that making a repository of isolated endemic strains is an urgent issue. By using endemic strains, serological assays can be optimized, which would support determination of the optimal positivity cut-off values of the IgM ELISA in northern Vietnam.

## Supporting information

S1 Checklist STROBE statement checklist. (DOCX)

S1 Fig Excluded cases with known diagnoses upon admission. (TIF)

S2 Fig Decision tree by classification and regression tree analysis to diagnose scrub typhus. ST: scrub typhus, AST: aspartate aminotransferase, HR: heart rate.
(TIF)

S3 Fig Receiver operating characteristic curve of ST diagnosis by IgM ELISA in the first blood samples. ROC: receiver operating characteristic.
(TIF)

S4 Fig Receiver operating characteristic curve of ST diagnosis by IgM ELISA in the second blood samples. ROC: receiver operating characteristic.
(TIF)

S5 Fig Phylogenetic tree of 47-kDa *HtrA* gene sequences of *O*. *tsutsugamushi* and *O*. *chuto*. GenBank accession number and strain name of all accessible sequences of the 47-kDa *HtrA* gene in GenBank on 29/10/2018 are indicated. The evolutionary history was inferred by using the maximum likelihood method based on the Hasegawa-Kishino-Yano model with a discrete gamma distribution with a bootstrap test of 1000 replicates. Bootstrap values higher than 70 were considered to be statistically significant and are shown next to the branches. The tree is drawn to scale, with branch lengths measured by the number of substitutions per site. All positions with less than 95% site coverage were eliminated. That is, fewer than 5% of alignment gaps, missing data, and ambiguous bases were allowed at any position. There were a total of 657 positions in the final dataset. Evolutionary analyses were conducted in MEGA6. Types of antigen with the highest reaction by immunoperoxidase assay are indicated with a bracket, if the patient was tested. Kp: Karp, Kt: Kato, Gm: Gilliam, Hi: Hirano/Kuroki, black circle: strains in the present study (GenBank accession numbers: LC431268-LC431284).
(TIF)

S1 Table Diagnosis category and pathogen identifiability. Diagnoses were extracted from medical charts and categorized by specific etiology and organ system. Pathogen identification status was based on medical charts, reflecting the available information at discharge by bedside staffs. i) HIV/AIDS status is the most prioritized condition to determine HIV/AIDS-related infection, which includes any organ system infections associated with HIV/AIDS. ii) Other local infection includes subacute thyroiditis, suppurative mastitis, septic arthritis, tonsillitis, local abscess and lymphadenitis. iii) Other systemic infection includes influenza, malaria, melioidosis, mumps, penicilliosis, rat-bite fever, and typhoid fever. iv) *Mycobacterium tuberculosis* infection is the most prioritized condition other than HIV/AIDS status to determine tuberculosis related diseases, which includes any organ system infections without HIV/AIDS. HIV/AIDS: human immunodeficiency virus/acquired immunodeficiency syndrome, NA: not applicable.
(XLSX)

S2 Table Maximum IgM/IgG titers by IIP among 50 scrub typhus cases with paired plasma samples in order of IgM ELISA OD value of the 2nd plasma samples with the blood sampling interval. Total number of observations: N = 50. Color code—red ID: PCR-positive (pink: only 56-kDa *tsa* gene-positive), yellow titer: IgM/IgG titer ≥ 4-fold increase (light yellow: IgM titer ≥ 320), blue OD value: ELISA-positive (pale blue: ELISA positive only by 2nd plasma). IIP: indirect immunoperoxidase assay, OD: optical density, *tsa*: type specific antigen, Gm: Gilliam, Kp: Karp, Kt: Kato, Ir: Irie/Kawasaki, Hi: Hirano/Kuroki, Sh: Shimokoshi, Rt: *Rickettsia typhi*, Rj: *R*. *japonica*, SFTS: severe fever with thrombocytopenia syndrome virus.
(XLSX)

S3 Table Comparison of the demographic and clinical characteristics between scrub typhus cases, ELISA-positive non-ST patients, and ELISA-negative non-ST patients. Total number of observations: N = 1127. * P values were calculated by comparing the three groups by Fisher's exact test for categorical variables, ANOVA for normally distributed continuous variables, and the Kruskal-Wallis test for other continuous variables. ** qSOFA score: quick sequential organ failure assessment score (range 0–3 points, with 1 point each for systolic hypotension [≤ 100mmHg], tachypnea [≥ 22 /min], or altered mental status [Glasgow coma scale < 15]). ST: scrub typhus, ELISA (+): positive results by enzyme-linked immunosorbent assay, PCR (-): negative results by polymerase chain reaction, SD: standard deviation, IQR: interquartile range, WBC: white blood cell, AST: aspartate transaminase, ALT: alanine transaminase, CRP: C-reactive protein, DOXY/AZM: doxycycline or azithromycin.
(XLSX)

S1 Dataset Dataset of the study. (XLSX)
