# Supplementary material for: Challenges in diagnosing scrub typhus among hospitalized patients with undifferentiated fever at a national tertiary hospital in northern Vietnam
Source: PLoS Negl Trop Dis. 2019 Dec 5;13(12):e0007928. doi: 10.1371/journal.pntd.0007928 (PMC6917290; doi:10.1371/journal.pntd.0007928)
Supplement: S1 Checklist — (DOCX) [file pntd.0007928.s001.docx]

STROBE Statement—checklist of items that should be included in reports of observational studies

|  | Item No. | Recommendation | Section / paragraph | Relevant text from manuscript |
| --- | --- | --- | --- | --- |
| **Title and abstract** | 1 | (*a*) Indicate the study’s design with a commonly used term in the title or the abstract | Abstract | An observational study at a tertiary hospital. |
|  |  | (*b*) Provide in the abstract an informative and balanced summary of what was done and what was found | Abstract | Please read the abstract. |
| Introduction | | | |  |
| Background/rationale | 2 | Explain the scientific background and rationale for the investigation being reported | 1^st^-3^rd^ paragraphs | The last sentences of the three paragraphs. |
| Objectives | 3 | State specific objectives, including any prespecified hypotheses | Last paragraph | Please read the paragraph. |
| Methods | | | |  |
| Study design | 4 | Present key elements of study design early in the paper | Study design, participants, and enrolment criteria | The 1^st^ sentence of the section (a prospective observational study). |
| Setting | 5 | Describe the setting, locations, and relevant dates, including periods of recruitment, exposure, follow-up, and data collection | Study design, participants, and enrolment criteria / Study procedures and data collection | The 1^st^-3^rd^ sentences of the former section and the whole section of Study procedures and data collection. |
| Participants | 6 | (*a*) Give the eligibility criteria, and the sources and methods of selection of participants. | Study design, participants, and enrolment criteria / Study procedures and data collection | The 3^rd^-5^th^ sentences of the former section and the whole section of Study procedures and data collection. |
| Variables | 7 | Clearly define all outcomes, exposures, predictors, potential confounders, and effect modifiers. Give diagnostic criteria, if applicable | Study procedures and data collection / Case definitions | All exposures were summarized in Table 1, and the diagnostic criteria was written in the latter section. |
| Data sources/ measurement | 8 | For each variable of interest, give sources of data and details of methods of assessment (measurement). Describe comparability of assessment methods if there is more than one group | Study procedures and data collection / Serological tests / PCR and phylogenetic analysis / Statistical analysis | Please read the whole sections. All the clinical information was collected from medical charts, and the diagnostic tests for scrub typhus were written in Serological tests and PCR and phylogenetic analysis. |
| Bias | 9 | Describe any efforts to address potential sources of bias | Study design, participants, and enrolment criteria / Serological tests / PCR and phylogenetic analysis / Case definition | In the four sections, we described that we enrolled almost all undifferentiated fever patients admitted in the department of infectious diseases through a year, used two serological and three PCR assays for confirming the diagnosis. |
| Study size | 10 | Explain how the study size was arrived at |  | Not estimated in advance. |
| Quantitative variables | 11 | Explain how quantitative variables were handled in the analyses. If applicable, describe which groupings were chosen and why | Statistical analysis | Whole the section describes how quantitative variables were handled. |
| Statistical methods | 12 | (*a*) Describe all statistical methods, including those used to control for confounding | Statistical analysis | Please read the section. |
|  |  | (*b*) Describe any methods used to examine subgroups and interactions |  | Not applicable. |
|  |  | (*c*) Explain how missing data were addressed | Statistical analysis / Table 2 | Missing data were imputed by multiple imputation using multivariate normal regression models. |
|  |  | (*d*) If applicable, describe analytical methods taking account of sampling strategy |  | Not applicable. |
|  |  | (*e*) Describe any sensitivity analyses |  | Not applicable. |
| Results | | | | |
| Participants | 13 | (a) Report numbers of individuals at each stage of study—eg numbers potentially eligible, examined for eligibility, confirmed eligible, included in the study, completing follow-up, and analysed | Investigation protocol, Fig 1, and S1 Fig | Please read whole the section and see Fig 1 and S1 Fig. |
|  |  | (b) Give reasons for non-participation at each stage | Investigation protocol, Fig 1, and S1 Fig | Please read the 2^nd^-3^rd^ sentences of the section and see Fig 1 and S1 Fig. |
|  |  | (c) Consider use of a flow diagram | Fig 1 | Please see Fig 1. |
| Descriptive data | 14 | (a) Give characteristics of study participants (eg demographic, clinical, social) and information on exposures and potential confounders | Demographic and clinical characteristics and Table 1 | Please read whole the section and see Table 1. |
|  |  | (b) Indicate number of participants with missing data for each variable of interest | Table 1 | Please see Table 1. |
| Outcome data | 15 | Report numbers of outcome events or summary measures | Investigation protocol, Fig 1, and Table 1 | Please read the last sentence of the section and see Fig 1 and Table 1. |
| Main results | 16 | (*a*) Give unadjusted estimates and, if applicable, confounder-adjusted estimates and their precision (eg, 95% confidence interval). Make clear which confounders were adjusted for and why they were included | Demographic and clinical characteristics, Table 1, and Table 2 | Please read the section and see Table 1 and Table 2. |
|  |  | (*b*) Report category boundaries when continuous variables were categorized | Table 1 | Please see Table 1. |
|  |  | (*c*) If relevant, consider translating estimates of relative risk into absolute risk for a meaningful time period |  | Not applicable. |
| Other analyses | 17 | Report other analyses done—eg analyses of subgroups and interactions, and sensitivity analyses |  | Not applicable. |
| Discussion | | | | |
| Key results | 18 | Summarise key results with reference to study objectives | 1^st^ paragraph | Please read the 1^st^ paragraph of Discussion. |
| Limitations | 19 | Discuss limitations of the study, taking into account sources of potential bias or imprecision. Discuss both direction and magnitude of any potential bias | 3^rd^ and 6^th^ paragraphs | Please read whole the former paragraph and the 1^st^-7^th^ sentences of the latter paragraph. |
| Interpretation | 20 | Give a cautious overall interpretation of results considering objectives, limitations, multiplicity of analyses, results from similar studies, and other relevant evidence | 6^th^ paragraph | Please read whole the paragraph. |
| Generalisability | 21 | Discuss the generalisability (external validity) of the study results | 4^th^ and 6^th^ paragraphs | The 7^th^-8^th^ sentences of the former paragraph and the 5^th^-7^th^ sentences of the latter paragraph. |
| Other information | |  | | |
| Funding | 22 | Give the source of funding and the role of the funders for the present study and, if applicable, for the original study on which the present article is based |  | Information transmitted during the submission process. |
