# Supplementary figures and images for: Challenges in diagnosing scrub typhus among hospitalized patients with undifferentiated fever at a national tertiary hospital in northern Vietnam
Source: PLoS Negl Trop Dis. 2019 Dec 5;13(12):e0007928. doi: 10.1371/journal.pntd.0007928 (PMC6917290; doi:10.1371/journal.pntd.0007928)

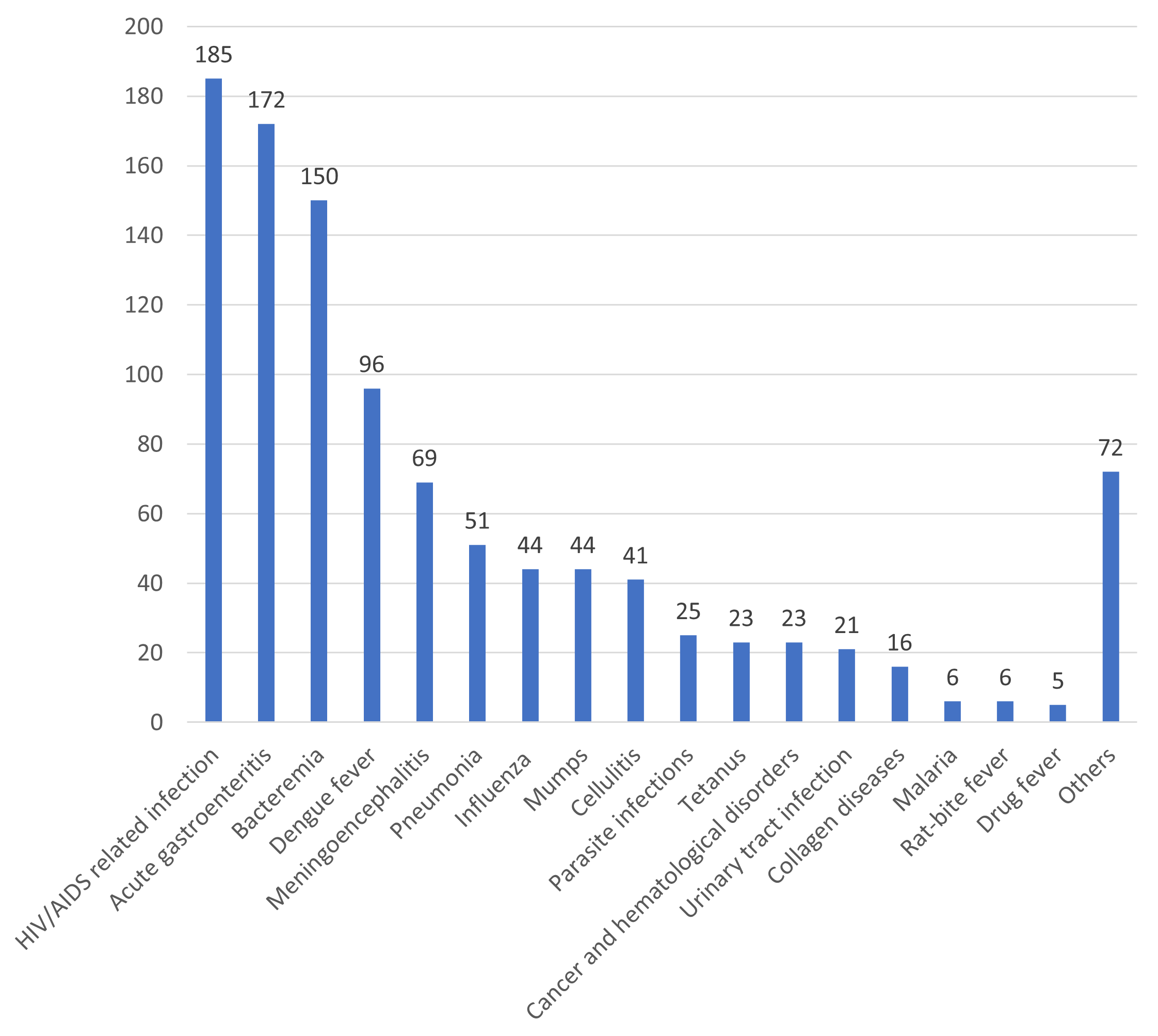

Supplement: S1 Fig — (TIF) [file pntd.0007928.s002.tif]

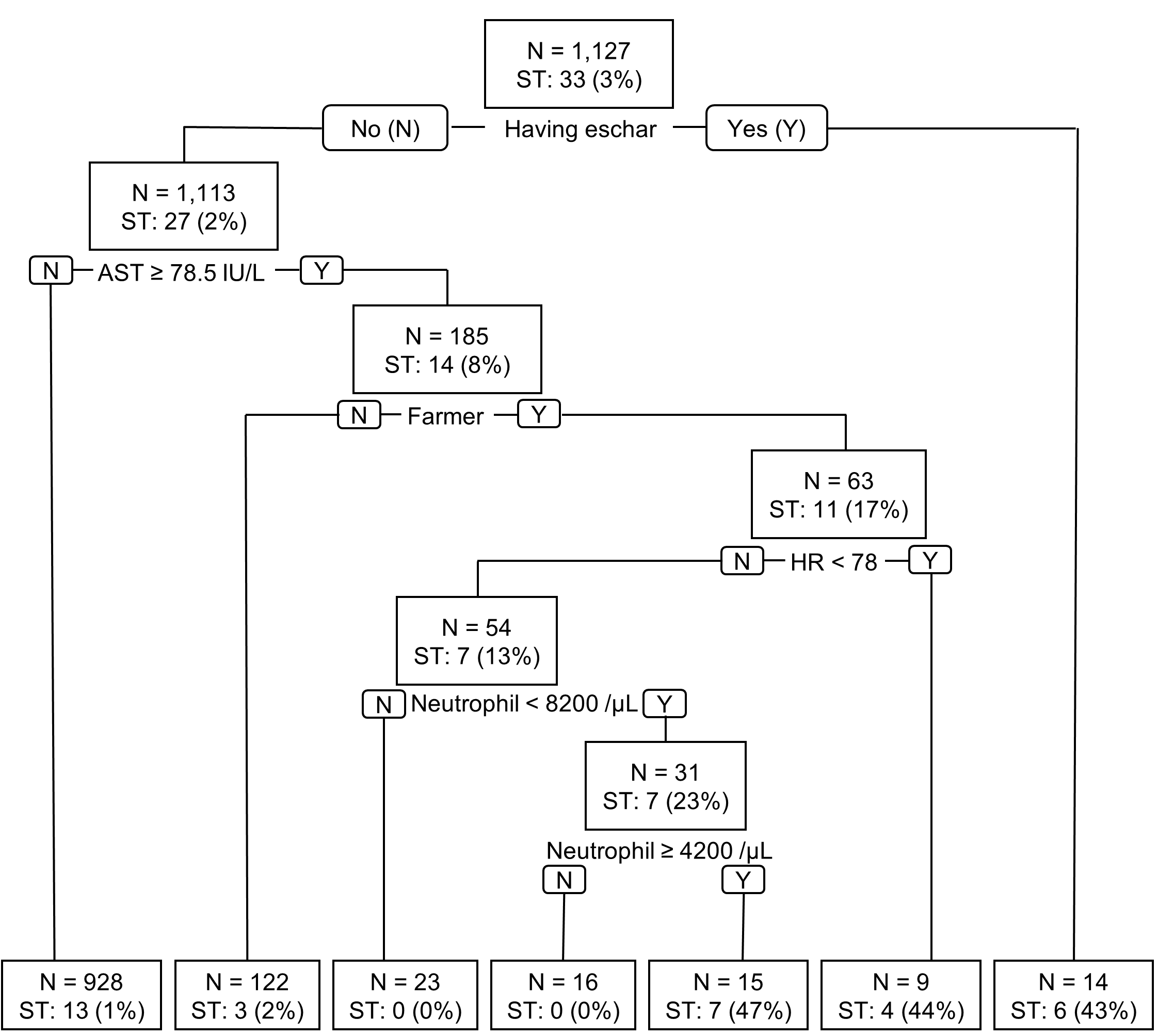

Supplement: S2 Fig — ST: scrub typhus, AST: aspartate aminotransferase, HR: heart rate. (TIF) [file pntd.0007928.s003.tif]

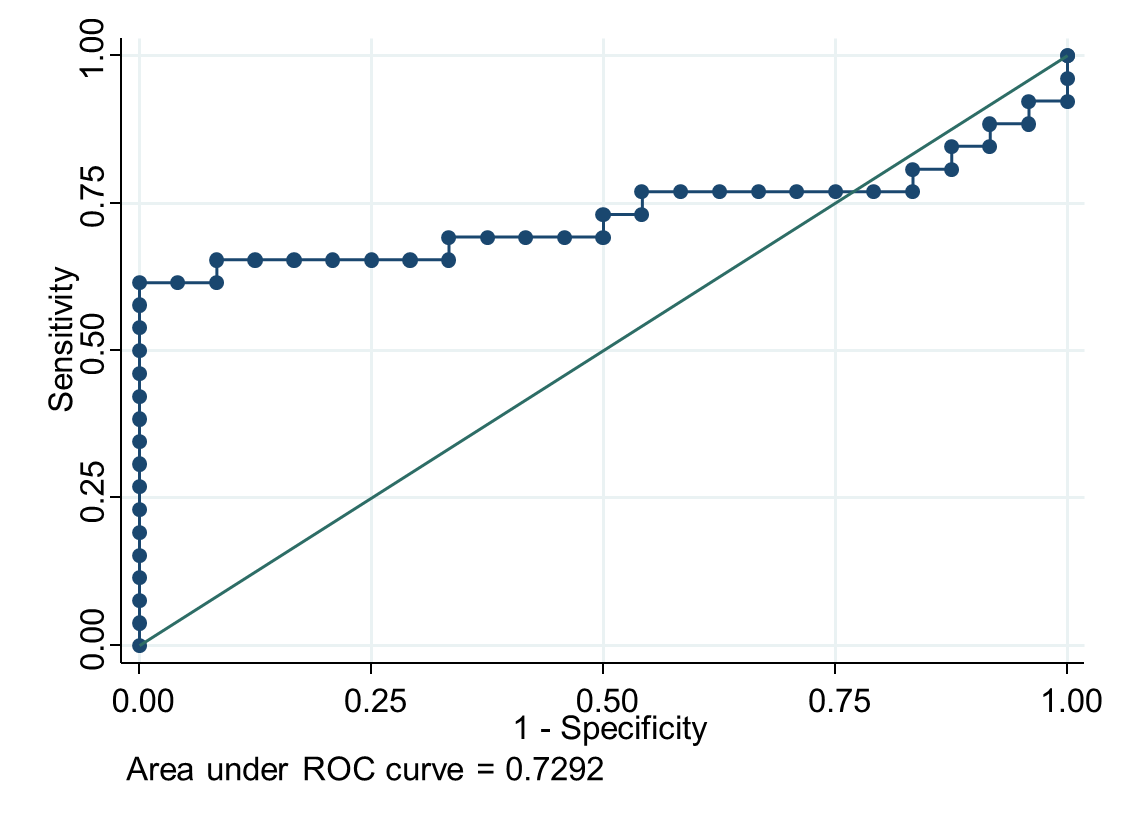

Supplement: S3 Fig — ROC: receiver operating characteristic. (TIF) [file pntd.0007928.s004.tif]

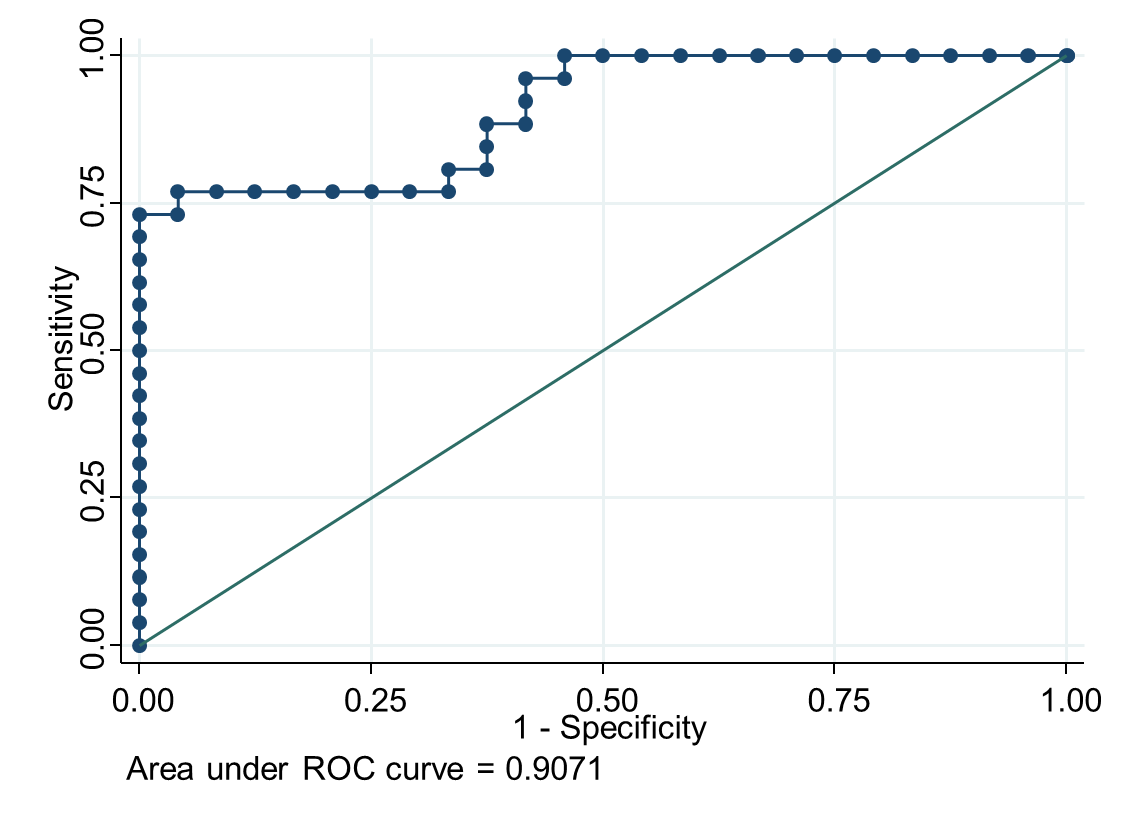

Supplement: S4 Fig — ROC: receiver operating characteristic. (TIF) [file pntd.0007928.s005.tif]

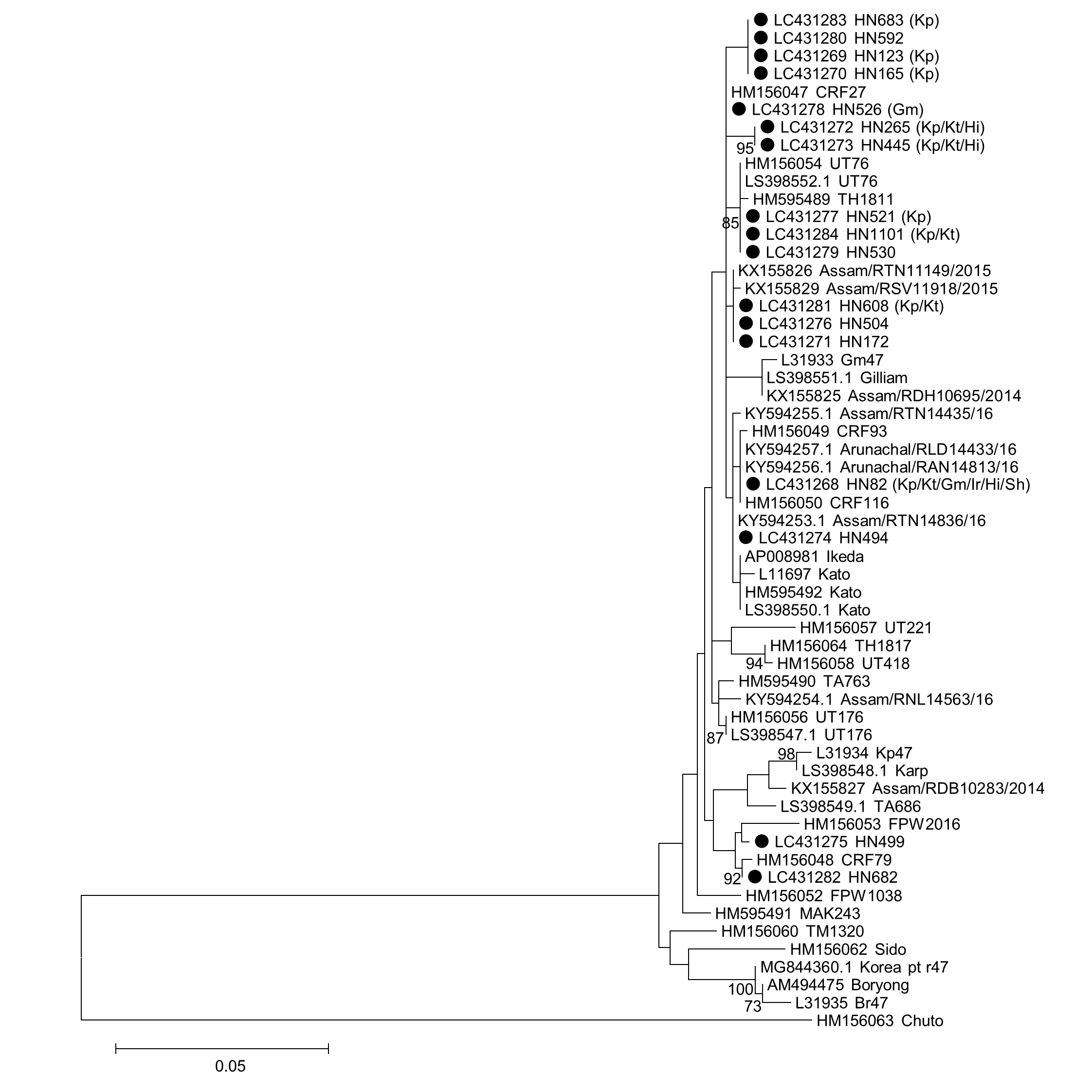

Supplement: S5 Fig — GenBank accession number and strain name of all accessible sequences of the 47-kDa HtrA gene in GenBank on 29/10/2018 are indicated. The evolutionary history was inferred by using the maximum likelihood method based on the Hasegawa-Kishino-Yano model with a discrete gamma distribution with a bootstrap test of 1000 replicates. Bootstrap values higher than 70 were considered to be statistically significant and are shown next to the branches. The tree is drawn to scale, with branch lengths measured by the number of substitutions per site. All positions with less than 95% site coverage were eliminated. That is, fewer than 5% of alignment gaps, missing data, and ambiguous bases were allowed at any position. There were a total of 657 positions in the final dataset. Evolutionary analyses were conducted in MEGA6. Types of antigen with the highest reaction by immunoperoxidase assay are indicated with a bracket, if the patient was tested. Kp: Karp, Kt: Kato, Gm: Gilliam, Hi: Hirano/Kuroki, black circle: strains in the present study (GenBank accession numbers: LC431268-LC431284). (TIF) [file pntd.0007928.s006.tif]
